# (*E*)-*N*′-(2-Chloro­benzyl­idene)-4-methoxy­benzohydrazide

**DOI:** 10.1107/S1600536809037854

**Published:** 2009-09-26

**Authors:** Wen-Dong Zhu, Su-Wei Chen

**Affiliations:** a College of Agriculture and Life Sciences, Ankang University, Ankang Shanxi 725000, People’s Republic of China

## Abstract

The mol­ecule of the title compound, C_15_H_13_ClN_2_O_2_, adopts an *E* geometry about the C=N bond. The dihedral angle between the two benzene rings is 62.7 (2)°. In the crystal structure, mol­ecules are linked through inter­molecular N—H⋯O hydrogen bonds, forming chains running along the *c* axis.

## Related literature

For the crystal structures of related hydrazone compounds, see: He & Liu (2005 ▶); Zhen & Han (2005 ▶); Fun *et al.* (2008 ▶); Qu & Cao (2009 ▶).
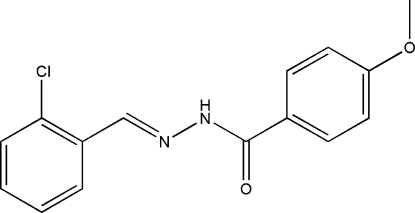


## Experimental

### 

#### Crystal data

C_15_H_13_ClN_2_O_2_
*M* *_r_* = 288.72
Monoclinic, 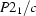

*a* = 11.5488 (9) Å
*b* = 13.4244 (10) Å
*c* = 9.6207 (7) Å
β = 107.873 (4)°
*V* = 1419.57 (18) Å^3^
*Z* = 4
Mo *K*α radiation
μ = 0.27 mm^−1^
*T* = 298 K
0.23 × 0.23 × 0.20 mm

#### Data collection

Bruker SMART CCD area-detector diffractometer
Absorption correction: multi-scan (*SADABS*; Bruker, 2001 ▶) *T*_min_ = 0.940, *T*_max_ = 0.948
8562 measured reflections
3084 independent reflections
2304 reflections with *I* > 2σ(*I*)
*R*_int_ = 0.022

#### Refinement

*R*[*F* ^2^ > 2σ(*F* ^2^)] = 0.046
*wR*(*F* ^2^) = 0.133
*S* = 1.02
3084 reflections
185 parameters
1 restraint
H atoms treated by a mixture of independent and constrained refinement
Δρ_max_ = 0.19 e Å^−3^
Δρ_min_ = −0.51 e Å^−3^

### 

Data collection: *SMART* (Bruker, 2007 ▶); cell refinement: *SAINT* (Bruker, 2007 ▶); data reduction: *SAINT*; program(s) used to solve structure: *SHELXTL* (Sheldrick, 2008 ▶); program(s) used to refine structure: *SHELXTL*; molecular graphics: *SHELXTL*; software used to prepare material for publication: *SHELXTL*.

## Supplementary Material

Crystal structure: contains datablocks global, I. DOI: 10.1107/S1600536809037854/wn2348sup1.cif

Structure factors: contains datablocks I. DOI: 10.1107/S1600536809037854/wn2348Isup2.hkl

Additional supplementary materials:  crystallographic information; 3D view; checkCIF report

## Figures and Tables

**Table 1:** Hydrogen-bond geometry (Å, °)

*D*—H⋯*A*	*D*—H	H⋯*A*	*D*⋯*A*	*D*—H⋯*A*
N2—H2⋯O1^i^	0.901 (10)	1.994 (12)	2.8717 (17)	165 (2)

Symmetry code: (i) 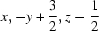.

**Fig. 1.**
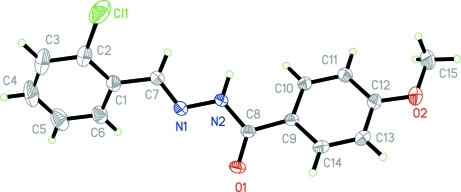
The molecular structure of the title compound with ellipsoids drawn at the 30% probability level. Hydrogen atoms are shown as spheres of arbitrary radius.

**Fig. 2.**
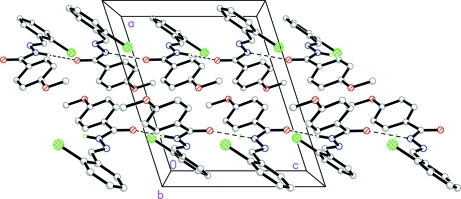
The molecular packing of the title compound, viewed along the b axis. Hydrogen bonds are drawn as dashed lines. Hydrogen atoms not involved in hydrogen bonding have been omitted.

## supplementary crystallographic information

### Comment

In recent years, the crystal structures of hydrazone compounds have attracted much attention (He & Liu, 2005; Zhen & Han, 2005; Fun *et al.*, 2008; Qu & Cao, 2009). In this paper, the new title compound (Fig. 1) is reported.

The molecule of the title compound adopts an *E* geometry about the C7═N1 bond. The dihedral angle between the C1-C6 and C9-C14 benzene rings is 62.7 (2)°.

In the crystal structure, molecules are linked through intermolecular N—H···O hydrogen bonds (Table 1) to form chains running along the *c* axis (Fig. 2).

### Experimental

Equimolar quantities of 2-chlorobenzaldehyde and 4-methoxybenzohydrazide were refluxed in methanol. Colorless block-shaped crystals were formed by slow evaporation of the solution in air.

### Refinement

H2 was located in a difference Fourier map and refined isotropically, with the N—H distance restrained to 0.90 (1) Å. The other H atoms were placed in idealized positions and constrained to ride on their parent atoms, with C—H distances of 0.93 and 0.96 Å, and with *U*_iso_(H) = k*U*_eq_(C), where k = 1.2 for Csp^2^ and 1.5 for methyl C.

### Crystal data

**Table:**

C_15_H_13_ClN_2_O_2_	*F*(000) = 600
*M**_r_* = 288.72	*D*_x_ = 1.351 Mg m^−^^3^
Monoclinic, *P*2_1_/*c*	Mo *K*α radiation, λ = 0.71073 Å
Hall symbol: -P 2ybc	Cell parameters from 2779 reflections
*a* = 11.5488 (9) Å	θ = 2.4–27.4°
*b* = 13.4244 (10) Å	µ = 0.27 mm^−^^1^
*c* = 9.6207 (7) Å	*T* = 298 K
β = 107.873 (4)°	Block, colorless
*V* = 1419.57 (18) Å^3^	0.23 × 0.23 × 0.20 mm
*Z* = 4	

### Data collection

**Table:**

Bruker SMART CCD area-detector diffractometer	3084 independent reflections
Radiation source: fine-focus sealed tube	2304 reflections with *I* > 2σ(*I*)
graphite	*R*_int_ = 0.022
ω scans	θ_max_ = 27.0°, θ_min_ = 1.9°
Absorption correction: multi-scan (*SADABS*; Bruker, 2001)	*h* = −14→12
*T*_min_ = 0.940, *T*_max_ = 0.948	*k* = −16→15
8562 measured reflections	*l* = −12→11

### Refinement

**Table:**

Refinement on *F*^2^	Primary atom site location: structure-invariant direct methods
Least-squares matrix: full	Secondary atom site location: difference Fourier map
*R*[*F*^2^ > 2σ(*F*^2^)] = 0.046	Hydrogen site location: inferred from neighbouring sites
*wR*(*F*^2^) = 0.133	H atoms treated by a mixture of independent and constrained refinement
*S* = 1.02	*w* = 1/[σ^2^(*F*_o_^2^) + (0.0675*P*)^2^ + 0.3322*P*] where *P* = (*F*_o_^2^ + 2*F*_c_^2^)/3
3084 reflections	(Δ/σ)_max_ = 0.001
185 parameters	Δρ_max_ = 0.19 e Å^−^^3^
1 restraint	Δρ_min_ = −0.50 e Å^−^^3^

### Special details

**Table:**

Geometry. All esds (except the esd in the dihedral angle between two l.s. planes) are estimated using the full covariance matrix. The cell esds are taken into account individually in the estimation of esds in distances, angles and torsion angles; correlations between esds in cell parameters are only used when they are defined by crystal symmetry. An approximate (isotropic) treatment of cell esds is used for estimating esds involving l.s. planes.
Refinement. Refinement of F^2^ against ALL reflections. The weighted R-factor wR and goodness of fit S are based on F^2^, conventional R-factors R are based on F, with F set to zero for negative F^2^. The threshold expression of F^2^ > 2sigma(F^2^) is used only for calculating R-factors(gt) etc. and is not relevant to the choice of reflections for refinement. R-factors based on F^2^ are statistically about twice as large as those based on F, and R- factors based on ALL data will be even larger.

### Fractional atomic coordinates and isotropic or equivalent isotropic displacement parameters (Å^2^)

**Table:**

	*x*	*y*	*z*	*U*_iso_*/*U*_eq_	
Cl1	0.23118 (7)	1.10791 (5)	0.47212 (9)	0.0924 (3)	
N1	0.19678 (13)	0.85010 (10)	0.71097 (14)	0.0399 (3)	
N2	0.24583 (13)	0.76562 (10)	0.67022 (14)	0.0403 (3)	
O1	0.29267 (12)	0.69949 (9)	0.89788 (12)	0.0479 (3)	
O2	0.46641 (14)	0.34135 (10)	0.58877 (16)	0.0628 (4)	
C1	0.12586 (16)	1.01500 (13)	0.65257 (19)	0.0447 (4)	
C2	0.14399 (19)	1.10494 (14)	0.5909 (3)	0.0583 (5)	
C3	0.0947 (2)	1.19280 (17)	0.6231 (3)	0.0813 (8)	
H3	0.1089	1.2525	0.5820	0.098*	
C4	0.0258 (3)	1.1916 (2)	0.7147 (4)	0.0884 (9)	
H4	−0.0073	1.2506	0.7359	0.106*	
C5	0.0045 (2)	1.1040 (2)	0.7763 (3)	0.0803 (8)	
H5	−0.0431	1.1037	0.8386	0.096*	
C6	0.05407 (18)	1.01637 (17)	0.7455 (2)	0.0567 (5)	
H6	0.0393	0.9572	0.7874	0.068*	
C7	0.18011 (16)	0.92262 (12)	0.62146 (18)	0.0413 (4)	
H7	0.2024	0.9168	0.5367	0.050*	
C8	0.29077 (15)	0.69279 (12)	0.77010 (17)	0.0370 (4)	
C9	0.33550 (15)	0.60262 (11)	0.71352 (17)	0.0369 (4)	
C10	0.29691 (16)	0.57429 (13)	0.56828 (18)	0.0432 (4)	
H10	0.2421	0.6143	0.5002	0.052*	
C11	0.33849 (17)	0.48743 (13)	0.52260 (19)	0.0469 (4)	
H11	0.3109	0.4692	0.4247	0.056*	
C12	0.42069 (17)	0.42791 (13)	0.6221 (2)	0.0460 (4)	
C13	0.4634 (2)	0.45714 (15)	0.7671 (2)	0.0586 (5)	
H13	0.5215	0.4188	0.8340	0.070*	
C14	0.42027 (19)	0.54236 (14)	0.81211 (19)	0.0530 (5)	
H14	0.4480	0.5603	0.9101	0.064*	
C15	0.4283 (2)	0.31064 (15)	0.4399 (3)	0.0627 (6)	
H15A	0.3420	0.3000	0.4082	0.094*	
H15B	0.4689	0.2498	0.4304	0.094*	
H15C	0.4484	0.3614	0.3809	0.094*	
H2	0.260 (2)	0.7642 (19)	0.5831 (15)	0.080*	

### Atomic displacement parameters (Å^2^)

**Table:**

	*U*^11^	*U*^22^	*U*^33^	*U*^12^	*U*^13^	*U*^23^
Cl1	0.0982 (5)	0.0642 (4)	0.1289 (6)	0.0040 (3)	0.0556 (5)	0.0324 (4)
N1	0.0465 (8)	0.0383 (7)	0.0371 (7)	0.0016 (6)	0.0163 (6)	−0.0040 (6)
N2	0.0580 (9)	0.0354 (7)	0.0325 (7)	0.0047 (6)	0.0210 (6)	0.0006 (6)
O1	0.0710 (8)	0.0470 (7)	0.0313 (6)	−0.0005 (6)	0.0239 (6)	0.0015 (5)
O2	0.0811 (10)	0.0446 (7)	0.0629 (9)	0.0194 (7)	0.0224 (7)	0.0000 (6)
C1	0.0416 (9)	0.0428 (9)	0.0427 (9)	0.0038 (7)	0.0026 (7)	−0.0065 (7)
C2	0.0513 (11)	0.0422 (10)	0.0734 (14)	0.0026 (8)	0.0073 (10)	−0.0011 (9)
C3	0.0709 (15)	0.0410 (12)	0.116 (2)	0.0081 (10)	0.0049 (15)	−0.0041 (12)
C4	0.0755 (17)	0.0652 (16)	0.114 (2)	0.0275 (13)	0.0131 (16)	−0.0280 (15)
C5	0.0658 (15)	0.090 (2)	0.0834 (17)	0.0276 (13)	0.0203 (13)	−0.0204 (14)
C6	0.0497 (11)	0.0618 (12)	0.0556 (11)	0.0110 (9)	0.0117 (9)	−0.0076 (9)
C7	0.0481 (9)	0.0388 (8)	0.0370 (8)	0.0014 (7)	0.0130 (7)	−0.0021 (7)
C8	0.0445 (9)	0.0373 (8)	0.0315 (8)	−0.0034 (7)	0.0149 (7)	0.0010 (6)
C9	0.0462 (9)	0.0358 (8)	0.0305 (8)	−0.0005 (7)	0.0144 (7)	0.0039 (6)
C10	0.0495 (10)	0.0426 (9)	0.0335 (8)	0.0077 (8)	0.0070 (7)	0.0011 (7)
C11	0.0557 (11)	0.0453 (9)	0.0364 (9)	0.0047 (8)	0.0092 (8)	−0.0058 (7)
C12	0.0553 (11)	0.0366 (8)	0.0487 (10)	0.0045 (8)	0.0199 (8)	0.0033 (7)
C13	0.0757 (14)	0.0529 (11)	0.0432 (10)	0.0211 (10)	0.0123 (9)	0.0116 (8)
C14	0.0746 (13)	0.0511 (11)	0.0301 (8)	0.0108 (9)	0.0114 (8)	0.0057 (8)
C15	0.0724 (14)	0.0442 (11)	0.0751 (14)	0.0017 (9)	0.0277 (11)	−0.0144 (10)

### Geometric parameters (Å, °)

**Table:**

Cl1—C2	1.740 (3)	C5—H5	0.9300
N1—C7	1.275 (2)	C6—H6	0.9300
N1—N2	1.3773 (19)	C7—H7	0.9300
N2—C8	1.357 (2)	C8—C9	1.483 (2)
N2—H2	0.901 (10)	C9—C10	1.383 (2)
O1—C8	1.2261 (19)	C9—C14	1.394 (2)
O2—C12	1.355 (2)	C10—C11	1.383 (2)
O2—C15	1.424 (3)	C10—H10	0.9300
C1—C2	1.389 (3)	C11—C12	1.378 (2)
C1—C6	1.394 (3)	C11—H11	0.9300
C1—C7	1.461 (2)	C12—C13	1.386 (3)
C2—C3	1.385 (3)	C13—C14	1.370 (3)
C3—C4	1.356 (4)	C13—H13	0.9300
C3—H3	0.9300	C14—H14	0.9300
C4—C5	1.373 (4)	C15—H15A	0.9600
C4—H4	0.9300	C15—H15B	0.9600
C5—C6	1.379 (3)	C15—H15C	0.9600
			
C7—N1—N2	115.26 (14)	O1—C8—C9	121.78 (14)
C8—N2—N1	119.53 (13)	N2—C8—C9	115.37 (13)
C8—N2—H2	120.3 (16)	C10—C9—C14	117.97 (15)
N1—N2—H2	119.3 (16)	C10—C9—C8	123.68 (14)
C12—O2—C15	117.63 (15)	C14—C9—C8	118.35 (14)
C2—C1—C6	117.37 (18)	C11—C10—C9	121.14 (16)
C2—C1—C7	121.33 (18)	C11—C10—H10	119.4
C6—C1—C7	121.29 (17)	C9—C10—H10	119.4
C3—C2—C1	121.2 (2)	C12—C11—C10	120.04 (16)
C3—C2—Cl1	119.16 (19)	C12—C11—H11	120.0
C1—C2—Cl1	119.59 (15)	C10—C11—H11	120.0
C4—C3—C2	119.9 (2)	O2—C12—C11	124.65 (17)
C4—C3—H3	120.1	O2—C12—C13	115.91 (16)
C2—C3—H3	120.1	C11—C12—C13	119.43 (16)
C3—C4—C5	120.6 (2)	C14—C13—C12	120.20 (17)
C3—C4—H4	119.7	C14—C13—H13	119.9
C5—C4—H4	119.7	C12—C13—H13	119.9
C4—C5—C6	119.8 (3)	C13—C14—C9	121.14 (17)
C4—C5—H5	120.1	C13—C14—H14	119.4
C6—C5—H5	120.1	C9—C14—H14	119.4
C5—C6—C1	121.1 (2)	O2—C15—H15A	109.5
C5—C6—H6	119.4	O2—C15—H15B	109.5
C1—C6—H6	119.4	H15A—C15—H15B	109.5
N1—C7—C1	119.69 (16)	O2—C15—H15C	109.5
N1—C7—H7	120.2	H15A—C15—H15C	109.5
C1—C7—H7	120.2	H15B—C15—H15C	109.5
O1—C8—N2	122.83 (15)		

### Hydrogen-bond geometry (Å, °)

**Table:**

*D*—H···*A*	*D*—H	H···*A*	*D*···*A*	*D*—H···*A*
N2—H2···O1^i^	0.90 (1)	1.99 (1)	2.8717 (17)	165 (2)

Symmetry codes: (i) *x*, −*y*+3/2, *z*−1/2.
